# Lipopolysaccharide-induced endotoxemia in corn oil-preloaded mice causes an extended course of lung injury and repair and pulmonary fibrosis: A translational mouse model of acute respiratory distress syndrome

**DOI:** 10.1371/journal.pone.0174327

**Published:** 2017-03-23

**Authors:** Chaomin Wu, Colin E. Evans, Zhiyu Dai, Xiaojia Huang, Xianming Zhang, Hua Jin, Guochang Hu, Yuanlin Song, You-Yang Zhao

**Affiliations:** 1 Department of Pharmacology, University of Illinois College of Medicine, Chicago, Illinois, United States of America; 2 Center for Lung and Vascular Biology, University of Illinois College of Medicine, Chicago, Illinois, United States of America; 3 Department of Pulmonary Disease, Qingpu Branch of Zhongshan Hospital, Fudan University, Shanghai, China; 4 Department of Physiology, Development and Neuroscience, School of Biological Sciences, University of Cambridge, Cambridge, United Kingdom; 5 Department of Anesthesiology, University of Illinois College of Medicine, Chicago, Illinois, United States of America; 6 Department of Pulmonary Medicine, Zhongshan Hospital, Fudan University, Shanghai, China; Chinese Academy of Sciences, CHINA

## Abstract

Acute respiratory distress syndrome (ARDS) is characterized by acute hypoxemia respiratory failure, bilateral pulmonary infiltrates, and pulmonary edema of non-cardiac origin. Effective treatments for ARDS patients may arise from experimental studies with translational mouse models of this disease that aim to delineate the mechanisms underlying the disease pathogenesis. Mouse models of ARDS, however, can be limited by their rapid progression from injured to recovery state, which is in contrast to the course of ARDS in humans. Furthermore, current mouse models of ARDS do not recapitulate certain prominent aspects of the pathogenesis of ARDS in humans. In this study, we developed an improved endotoxemic mouse model of ARDS resembling many features of clinical ARDS including extended courses of injury and recovery as well as development of fibrosis following i.p. injection of lipopolysaccharide (LPS) to corn oil-preloaded mice. Compared with mice receiving LPS alone, those receiving corn oil and LPS exhibited extended course of lung injury and repair that occurred over a period of >2 weeks instead of 3–5days. Importantly, LPS challenge of corn oil-preloaded mice resulted in pulmonary fibrosis during the repair phase as often seen in ARDS patients. In summary, this simple novel mouse model of ARDS could represent a valuable experimental tool to elucidate mechanisms that regulate lung injury and repair in ARDS patients.

## Introduction

Acute respiratory distress syndrome (ARDS) remains a major cause of mortality in intensive care units worldwide [1–3]. The triggers of ARDS can be classified as direct (e.g. pneumonia, inhalation injury, aspiration of gastric contents, pulmonary contusion, or reperfusion pulmonary edema) or indirect (e.g. sepsis, burn, major trauma with shock, multiple blood transfusions, or acute pancreatitis) [1, 2, 4]. ARDS in humans is defined by a list of clinical parameters including diffuse alveolar damage and reduced pulmonary oxygenation [1, 2, 5, 6]. Human ARDS develops over a ≤7day period; in this time frame, the initial exudative/inflammatory stage is followed by a proliferative tissue repair stage; and after 3 weeks, the repair phase can culminate in pulmonary fibrosis (i.e. in two-thirds of ARDS patients) [7–9]. Although the incidence and mortality of ARDS has declined over recent years [3, 10], the mechanisms that regulate the pathogenesis and resolution of ARDS are incompletely understood, and there are still no effective pharmacological or cell-based treatments for this disease.

Mouse models of sepsis-induced inflammatory lung injury can be used to improve understanding of the mechanisms that control sepsis-induced lung injury and subsequent repair [4, 11]. It is therefore desirable for such mouse models to recapitulate the pathogenesis of human ARDS as closely as possible. Features of human ARDS also shown in animal models of ARDS have been documented in an American Thoracic Society workshop report [12]. Mouse models are often confounded, however, by the brief period of time over which recovery from injury subsequently occurs (i.e. usually between 2–4 days post-sepsis challenge), and the absence of post-sepsis pulmonary fibrosis. Endotoxemia induced by i.p. LPS is a widely used experimental model of sepsis-induced ARDS. This conventional model causes lung injury within hours after LPS challenge, which peaks within 24–36h followed by a rapid recovery phase over the next 2–3 days [13–17]. The primary aim of this study was to determine whether the course of LPS-induced lung injury and subsequent repair could be extended to a length comparable to that commonly seen in ARDS patients and to determine whether a mouse model with extended lung injury and repair will better recapitulate the pathogenesis of ARDS seen in patients including development of pulmonary fibrosis. The model we present here provides a means of studying mechanisms that regulate ARDS pathogenesis and resolution in mice.

## Materials and methods

### Reagents

Albumin, anti-vWF antibody, 5-bromo-2-deoxyuridine (BrdU), chloramine Ttrihydrate, corn oil, Ehrlich’s reagent, Evans blue dye, formamide, hematoxylin and eosin (H&E), Hexadecyl trimethylammonium bromide, hydroxyproline, O-dianisidine dihydrochloride, trichrome kit, and trichloroacetic acid were purchase from Sigma-Aldrich (St Louis, MO). LPS from E. coli 055: B5 was obtained from Santa Cruz Biotechnology, Inc. (Dallas, TX). RNeasy Mini kit, and DNase I were purchased from Qiagen USA (Germantown, MD). In Situ Cell Death Detection Kit, and SYBR Green master mix were obtained from Roche Diagnostics (Indianapolis, IN). Anti-BrdU antibody was purchased from BD Biosciences (San Jose, CA). DAPI, Alexa FITC-conjugated or Texas Red-conjugated secondary antibodies, and Trizol reagent were obtained from Thermo Fisher Scientific (Waltham, MA).

### Mice

C57BL/6 mice (12 to 14 week old, male and female) were used for the study. All mice were housed in the Association for Assessment and Accreditation of Laboratory Animal Care (AAALAC)-accredited animal facility at the University of Illinois at Chicago according to National Institutes of Health guidelines. All animal experiments were performed in accordance with protocols approved by the University of Illinois at Chicago Animal Care and Use Committee. After LPS administration, the mice were monitored 4 times a day for the first 5 days, and then once a day until end of the study. When performing retro-orbital injection of EBA, the mice were anesthetized with isofluorane. The mice were euthanized for tissue collection under anesthesia with Ketamine/Xylazine.

### Endotoxemic mouse models of lung injury induced by corn oil/LPS or LPS alone

Mice were administrated with corn oil (12ml/kg body weight, i.p.) every 48 hours for three times, and then with LPS (4mg/kg, i.p.) at 5 days post-last injection of corn oil. PBS was administered 5 days after corn oil administration as a control. Another cohort of mice received LPS alone (4mg/kg, i.p.).

### Vascular permeability assay

The Evans blue-conjugated albumin (EBA) extravasation assay was performed as previously described [18]. Briefly, EBA (20mg/kg) was retro-orbitally injected into mice 30 minutes before tissue collection. Lungs were perfused free of blood with PBS, blotted dry, and weighed. Lung tissue was then homogenized in 0.5ml PBS and incubated with 1ml formamide at 60°C for 18 hours. The homogenate was centrifuged at 5,000g for 30 minutes. Optical density of the supernatant was determined at 620nm and 740nm. The extravasated EBA in lung homogenate was expressed as mg of Evans blue dye per g lung tissue.

### Calculation of lung wet/dry weight ratio

Following measurement of the lung weight immediately after collection (wet weight), lungs were dried in the oven at 65°C for 72 hours and dry lung weight was measured for determination of lung wet/dry weight ratio.

### Myeloperoxidase assay

Myeloperoxidase (MPO) activity was measured as previously described [18]. Briefly, lung tissues free of blood were collected following perfusion with PBS and homogenized in 50mM phosphate buffer. Homogenates were centrifuged at 15,000g for 20 minutes at 4°C. Thereafter the pellets were resuspended in phosphate buffer containing 0.5% hexadecyl trimethylammonium bromide and subjected to a cycle of freezing and thawing. Subsequently the homogenates were centrifuged again at 15,000g for 20 minutes at 4°C. Following addition of 34μl of sample to 10μl of O-dianisidine dihydrochloride (O-DHC, 16.7mg/ml) and 50μl of H_2_O_2_ (0.015% v/v) in 1ml of phosphate buffer, absorbance was measured at 460nm every 20 seconds for 3 minutes and results were presented as △OD_460_/ min/g lung tissue.

### Quantitative real time RT-PCR analysis

Total RNA was isolated using an RNeasy Mini kit including DNase I digestion enzyme. Following reverse transcription, quantitative real-time PCR analysis was performed using the ABI ViiA^TM^ 7 real-time PCR system (Thermo Fisher Scientific) with SYBR Green master mix. The primers used for analysis were listed as following. mouse TNF- a, 5’-ATGCTGGGACAGTGACCTGG-3’ and 5’- CCTTGATGGTGGTGCATGAG-3’; mouse IL-6, 5’-TCCAGTTGCCTTCTTGGGACTG-3’and 5’-AGCCTCCGACTTGTGAAGTGGT-3’; mouse ICAM-1, 5’-GTCTCGGAAGGGAGCCAAGTA-3’ and 5’-CTCGACGCCGCTCAGAAGAA-3’; mouse iNOS, 5’-ACATCAGGTCGGCCATCACT-3’and 5’-CGTACCGGATGAGCTGTGAATT-3’; mouse IL-1β, 5’-AACCTGCTGGTGTGTGACGTTC-3’ and 5’-CAGCACGAGGCTTTTTTGTTGT-3’; mouse cyclin A2, 5’-AATGCAGCTGTCTCTTTACCCGCA- 3’ and 5’-CCTCCATTTCCCTAAGCTACGTGT- 3’; mouse cyclin B1, 5’-TGAACCAGAGGTGGAACTTGCTGA- 3’ and 5’-AGATGTTTCCATCGGGCTTGGAGA-3’; and mouse Cdc25C, 5’-TGAAGCATCTGGGCAGTCCCATTA-3’ and 5’-GGCAGCACACACACCTTTGAGAAA-3’. Gene expression was normalized to mouse Cyclophilin as previously described [19].

### Cell proliferation and cell apoptosis assay

Cell Proliferation Assay was performed as previously described [18]. Briefly, 5-bromo-2-deoxyuridine (BrdU) was administered 6 hours prior to tissue collection (75mg/kg, i.p.). Mouse lung cryosections (5μm) were stained overnight with anti-BrdU (1:5 dilution, BD Biosciences, USA) at 4°C then incubated with Alexa FITC-conjugated secondary antibody (1:200 dilution, Life Technologies, USA) for 30 minutes at room temperature. Lung vascular endothelial cells were immunostained with anti-vWF (1:300 dilution) and anti-CD31 (1:40 dilution) antibodies for 1 hour at room temperature. Sections were incubated with Texas Red-conjugated secondary antibody (1:700 dilution) for 30 minutes at room temperature. The nuclei were counterstained with DAPI. Two sections per lung tissue and 10 fields (20X) per section were quantified blindly.

Apoptosis was assessed using the In Situ Cell Death Detection Kit according to manufacturer’s instructions and anti-vWF and anti-CD31 antibodies were used to immunostain endothelial cells (ECs) as described above. Two sections per lung tissue and 10 fields (20X) per section were quantified blindly.

### Histological analysis

Left lung lobes were inflated to 20cm H_2_O transpulmonary pressure with 10% neutral buffered formalin and fixed in 10% formalin for overnight. Fixed lungs were embedded in paraffin, sectioned at 5μm thickness, and stained with either hematoxylin and eosin (H&E), or trichrome using standard laboratory protocols [13, 20].

### Hydroxyproline assay

Left lung lobes were removed and immediately frozen on dry ice then stored at -70°C until processing for assessment of hydroxyproline content according to established laboratory protocols. Briefly, lungs were homogenized in 1ml of PBS, then 62.5μl of 50% trichloroacetic acid was added to the homogenate followed by incubation of the samples on ice for 20 minutes. Thereafter, the samples were spun down for 30 minutes at 5000g and 4°C, then 500μl of 12N HCI was added to the pellet in a glass tube. Samples were then charred at 110°C for 14–18 hours in a fume hood. The dried pellet was reconstituted with 1ml of deionized water, then 200μl of each sample was added to 500μl of chloramine T solution (1.4% chloramine T in 0.5M sodium acetate and 10% isopropanol) and incubated for 20 minutes at room temperature. Thereafter, 500μl of Ehrlich’s reagent (1M *p*-dimethylaminobenzaldehyde in 70% isopropanol / 30% perchloric acid) was added and the sample incubated for 15 minutes at 65°C. The absorbance of each sample was then measured at 550nm. Standard curves for the experiment were generated using known concentrations of hydroxyproline.

### Statistical analysis

Differences between groups were assessed by Student’s t-tests (for paired comparisons) or by ANOVA with Bonferroni correction (for multiple comparisons). P values of less than 0.05 were considered statistically significant. All statistical analyses were performed using Prism 5.01 software (GraphPad, USA).

## Results

### Extended course of lung vascular injury and recovery in oil/LPS-challenged mice

**Fig 1:**
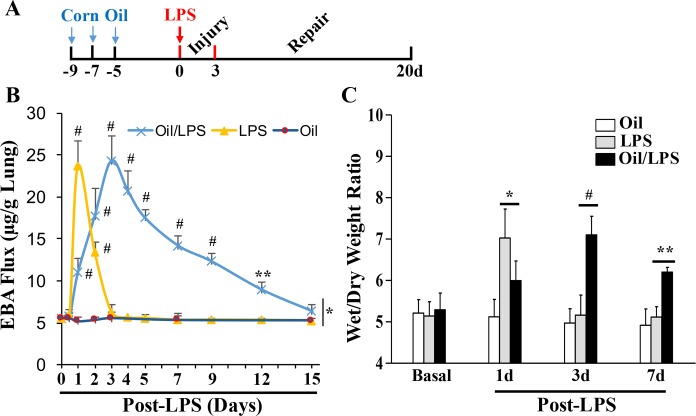
Extended course of lung vascular injury and recovery in corn oil/LPS-challenged mice. (**A**) The oil/LPS model: Corn oil was administered i.p. at 12ml/kg every 48h for 3 times. Five days after the last injection of corn oil (i.e., waiting for 5 days to avoid any potential effects from corn oil administration), the mice were challenged with. (**B**) Time course of lung vascular permeability assessed by EBA extravasation assay. The data demonstrated sustained lung vascular leakiness in corn oil/LPS-challenged mice compared to oil-free mice challenged with LPS. *, P < 0.05. **, P < 0.01; #, P < 0.001 versus basal with or without oil, respectively. Corn oil administration per se didn’t induce lung vascular injury. (**C**) The lung wet/dry weight ratio was determined to assess pulmonary edema. Oil-free mice exhibited marked lung edema at 1 day post-LPS challenge and returned to basal level at 3 days post-LPS. However, the mice pre-administered with corn oil exhibited lung edema at 3 and 7 days post-LPS challenge. All data are expressed as mean ± SD (n = 4/group). *, P < 0.05; **, P < 0.01; #, P < 0.001.

We established a modified endotoxemia mouse model by pre-administration of corn oil to mice followed by LPS challenge (4mg/kg, i.p.) (**Fig 1A**). To determine alterations in lung vascular permeability, we employed the EBA assay to assess pulmonary transvascular flux of Evans blue-conjugated albumin [14, 15]. As shown in **Fig 1B** in oil-free mice, we observed a marked increase in EBA flux that peaked at 24 hours post-LPS challenge (4mg/kg, i.p.) and returned to baseline at 72 hours post-LPS. In mice pre-administered with corn oil, however, EBA flux reached a similar maximum level at 72 hours post-LPS challenge, and did not recover to basal values until 15 days post-LPS, indicating that the addition of corn oil extends the time course of LPS-induced vascular injury and the subsequent recovery processes. Adminstration of corn oil per se did not induce lung vascular injury at baseline. Levels of lung edema were also determined by measurement of lung wet/dry weight ratios (**Fig 1C**). While wet/dry weight ratios were greater in oil-free mice compared with oil-treated mice at 24 hours post-LPS, these ratios were greater in oil-treated mice compared with oil-free mice at both 3 and 7 days post-LPS. In oil-free mice, wet/dry weight ratios returned to baseline at 3 days post-LPS challenge. Together, these data demonstrate that it takes 3 days to reach maximal lung injury in oil/LPS-challenged mice compared with 24 hours in mice challenged with LPS alone, and that it takes about 2 weeks for oil/LPS-challenged mice to recover compared with 2 days for mice challenged with LPS alone.

### Extended time course of lung inflammation and subsequent resolution in oil/LPS-challenged mice

**Fig 2:**
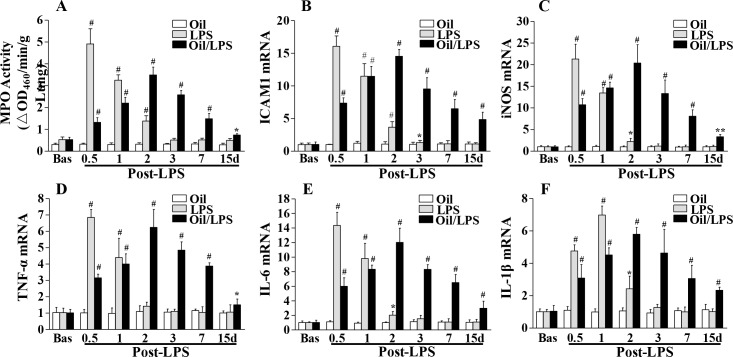
Extended course of lung inflammation and subsequent resolution in corn oil/LPS-treated mice. (**A**) Time course of lung MPO activity. At indicated times, lungs were collected after PBS perfusion free of blood for MPO activity assay. (**B**-**F**) QRT-PCR analyses of expression of proinflammatory genes in mouse lungs. The elevated expression of proinflammatory genes in oil-free mice decreased to basal levels at 3 days post-LPS challenge whereas it remained elevated at 15 days post-LPS in oil/LPS-challenged mice. Corn oil administration without LPS challenge didn’t induce any inflammatory response. All data are expressed as mean ± SD (n = 4/group). *, P < 0.05; **, P < 0.01; #, P < 0.001 versus basal with or without oil, respectively.

Next we assayed whether the levels of lung inflammation would be similarly affected by the addition of corn oil to LPS-treated mice, by measuring MPO activity, an indicator of neutrophil infiltration [14, 15]. In oil-free mice, lung MPO activity was markedly increased at 12 hours post-LPS challenge, and returned to basal levels at 72 hours post-LPS (**Fig 2A**), which is a pattern of change that is commonly seen in i.p. LPS-induced models of acute lung injury [14, 15]. In oil-loaded mice, however, MPO activity gradually increased and peaked at 48 hours post-LPS, and did not return to basal levels even at 15 days after LPS challenge (**Fig 2A**). As shown in **Fig 2B–2F**, levels of 5 proinflammatory factors (TNF-a, IL-1β, IL-6, ICAM-1, and iNOS) reached their peaks at 12 or 24 hours post-LPS administration in the lungs of mice without oil treatment, and these levels all returned to baseline at 72 hours post-LPS challenge. In the lungs of oil-loaded mice, however, the expression levels of these genes all peaked at 48 hours post-LPS challenge, and remained elevated at 15 days post-LPS challenge. We didn’t observe any inflammatory response in oil-loaded mice without LPS challenge.

**Fig 3:**
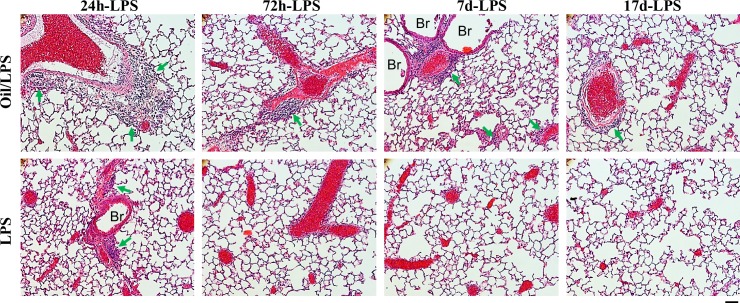
Histological analysis. Representative micrographs of H&E-stained mouse lung sections. At 24 hours post-LPS challenge, leukocyte sequestration was evident in mouse lungs of both LPS and oil/LPS groups. At 72 hours post-LPS challenge, leukocyte infiltration was minimal in lungs of oil-free mice but remained markedly elevated in lungs of corn oil-loaded mice. Even at 17 days post-LPS challenge, leukocyte sequestration in mouse lungs was still prominent in oil/LPS mice, indicating persistent lung inflammation in oil/LPS mice over 2 weeks. Arrows indicate leukocyte infiltration. Red cells are red blood cells. Scale bar, 50μm.

Histological assesment of lung sections taken from LPS-challenged and oil/LPS-challenged mice revealed similar findings (**Fig 3**). Neutrophil sequestration appeared greatest at 24 hours post-LPS in oil/LPS-challenged mice and persisted even at day 17 post-LPS, whereas few leukocytes were seen in lung sections at 72 hours post-LPS challenge in oil-free mice. These results suggest that addition of corn oil to LPS-induced endotoxemia delays the progression to peak lung inflammation, as well as substantially extends the subsequent restorative programs that culminate in resolution of lung inflammation.

### Persistent apoptosis in lungs of oil/LPS-challenged mice

**Fig 4:**
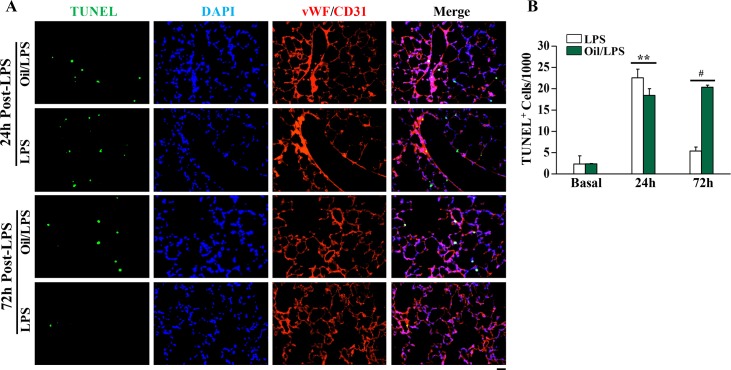
Sustained apoptosis in lungs of oil/LPS-challenged mice. (**A**) Representative micrographs of TUNEL-stained lung sections demonstrating marked increase of apoptosis at 24 and 72 hours post-LPS challenge in oil-loaded mice whereas apoptosis was prominent at 24 hours but minimal at 72 hours post-LPS in oil-free mice. In Situ Detection of Cell Death kit was employed to immunostain apoptotic cells (green) while anti-vWF and -CD31 antibodies (red) were used to co-immunostain vascular ECs. Nuclei were counterstained with DAPI (blue). Scale bar, 50μm. (**B**) Quantification of apoptosis. Data are expressed as mean ± SD (n = 4/group). **, *P* < 0.01; #, P < 0.001.

As seen in the acute phase of ARDS in humans, LPS-induced vascular injury in mice is characterized by increased vascular permeability and accompanying endothelial cell apoptosis [21]. We therefore assessed this cellular response in oil/LPS-challenged mice compared with LPS-challenged mice (**Fig 4**). At 24 hours post-LPS challenge, there was marked increases of apoptosis (mainly endothelial cells) in lung sections taken from oil-loaded and oil-free mice. At 72 hours post-LPS challenge, however, the level of apoptosis in lung sections of oil/LPS-challenged mice remained elevated, whereas fewer apoptotic cells were present in lung sections of mice challenged with LPS alone.

### Sustained reparative endothelial proliferation in oil/LPS-challenged mice

**Fig 5:**
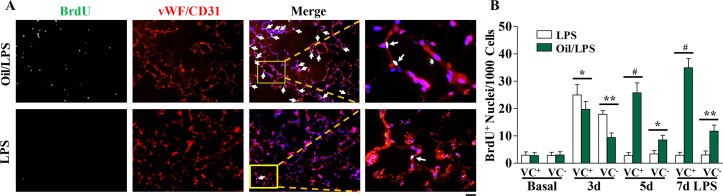
Cell proliferation in mouse lungs. (**A**) Representative micrographs of immunofluorescent staining. Lung tissues were collected at indicated times post-LPS challenge, sectioned and immunostained with anti-BrdU (green) antibody. Lung ECs were immunostained with anti-vWF and CD31 antibodies (red). Nuclei were counterstained with DAPI (blue). Arrows indicate proliferating EC. Scale bar, 50 μm. (**B**) Quantification of BrdU-positive ECs (vWF^+^ or CD31^+^) and non-ECs (vWF^-^ or CD31^-^) in mouse lungs. Data are expressed as mean ± SD (n = 4/group). *, P < 0.05; **, P < 0.01; #, P < 0.001. VC^+^, vWF^+^ or CD31^+^ ECs; VC^-^, vWF^-^ or CD31^-^ non-ECs.

**Fig 6:**
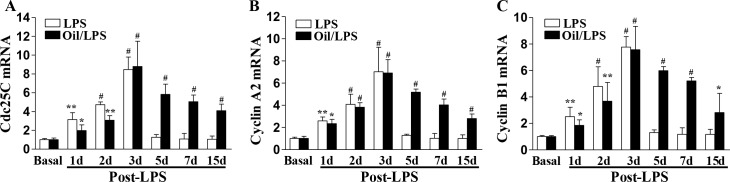
Expression of cell cycle genes in mouse lungs. At designated times post-LPS challenge, lungs were collected for RNA isolation and QRT-PCR analysis of Cdc25C, Cyclin A2, and Cyclin B1 mRNA. Data are expressed as mean ± SD (n = 4/group). *, P < 0.05; **, P < 0.01; #, P < 0.001 versus basal with or without corn oil, respectively.

It has been shown that EC proliferation plays an important role in endothelial repair after inflammatory lung injury following sepsis challenge [13, 15]. To determine cell proliferation in mouse lungs, mice were administered with BrdU at 6 hours prior to tissue collection and proliferating cells were detected by anti-BrdU immunostaining. As we reported previously in mice challenged with LPS alone, EC proliferation was markedly increased at 3 days post-LPS challenge and returned to baseline at day 5. (**Fig 5**). Also shown in **Fig 5**, EC proliferation was markedly increased in oil/LPS-challenged mice at day 3, post-LPS challenge and peaked at day 7. Although the proliferating cells were predominantly ECs, we also observed a marked increase in proliferation of non-ECs in oil/LPS mice at these times. However, proliferation of non-ECs returned to baseline in oil-free mice at day 5 and 7post-LPS challenge. Consistently, expression of genes essential for cell cycle progression was markedly induced and peaked at 72h post-LPS challenge in both oil/LPS-challenged mice and LPS-challenged mice (**Fig 6**). However, the expression of these cell cycle regulators was persistently elevated for at least within 2 weeks post-LPS challenge in oil/LPS-challenged mice, whereas levels returned to baseline at day 5 post-LPS challenge in oil-free LPS-challenged mice (**Fig 6**). These observations demonstrate that corn oil can be used to extend the molecular and cellular processes that control not only pulmonary (including endothelial) injury, but also pulmonary (including endothelial) repair.

### Prominent fibrosis in oil/LPS-challenged mice

**Fig 7:**
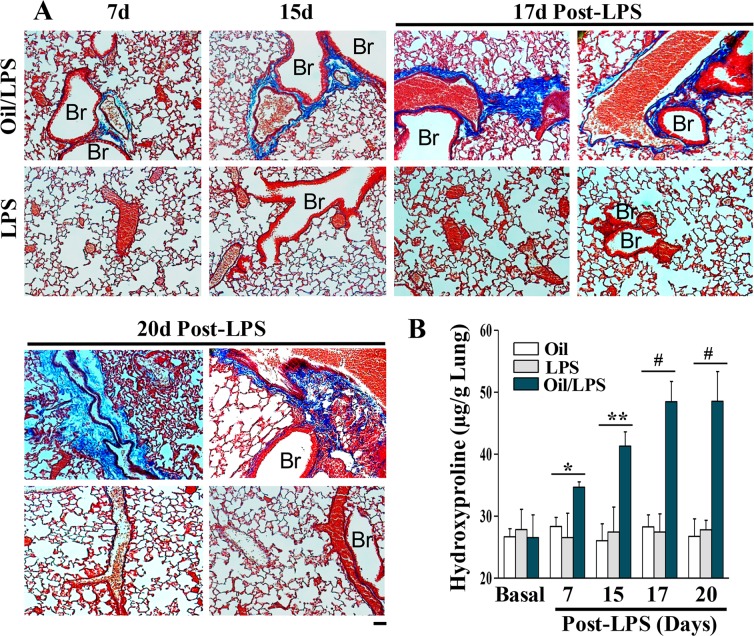
Prominent fibrosis in lungs of oil/LPS-challenged mice. (**A**) Representative micrographs of trichrome staining. Mouse lungs were collected at the indicated times following LPS challenge for trichrome staining. Fibrosis (blue) was seen in the adventitia of vessels at 7days post-LPS in oil-loaded mice, which was markedly increased at day 15 and after. At day 17 and 20 post-LPS challenge, fibrosis was also evident in the interstitial tissue and peribronchial regions besides the adventitia. However, there was no fibrosis in lungs of mice challenged with LPS alone. Br, bronchiole. (**B**) Quantification of lung fibrosis by hydroxyproline assay. Lung tissues were collected at the indicated times for hydroxyproline assay. Data are expressed as mean ± SD (n = 4/group). *, P < 0.05; **, P < 0.01; #, P < 0.001.

While human ARDS results in the manifestation of pulmonary fibrosis after recovery, mouse models of endotoxemic lung injury have not yet demonstrated clear evidence of post-injury fibrotic lung. In the oil-free mice at various times post-LPS challenge, there was no evidence of lung fibrosis as assessed by trichrome staining (**Fig 7A**), and lung hydroxyproline (**Fig 7B**). In corn oil/LPS-treated mice, however, we observed some evidence of fibrosis development as early as 7 days post-LPS, which progressed and peaked at day 17 post-LPS challenge and remained elevated at day 20 post-LPS (**Fig 7A** and **7B**). Intriguingly, fibrosis was initially limited in the adventitia of pulmonary vessels (e.g., day 7 and day 15) and then developed in the interstitial tissue around the vessels at days 17 and 20, as well as in peribronchial tissue to a lesser extent (**Fig 7A**).

## Discussion

Here we introduce a modified version of the conventional LPS-induced mouse model of ARDS that induces extended phases of post-sepsis injury and repair along with development of pulmonary fibrosis during the extended recovery phase as seen in ARDS patients. In mice pre-administered with corn oil, LPS-induced inflammatory lung injury peaked at 72 hours post-challenge and did not completely resolve until day 17–20 post-challenge. This extended course of lung repair resembles more closely the course of ARDS recovery in patients, which normally takes 2–4 weeks. Translational mouse models that better replicate the complex pathobiology of ARDS in man could lead to the identification of pathways that regulate human lung injury and subsequent repair, which could in turn be targeted for potential clinical benefit.

The lung inflammatory response in mouse models of ARDS that are triggered by i.p. LPS alone commonly peaks in as little as 4–12 hours post-LPS, and fully recovers within 3 days [14, 15, 22]. The pulmonary inflammatory response in human ARDS, however, is most intense for the initial 3 days following disease onset [23, 24]. In our corn oil/LPS model, pulmonary neutrophil sequestration as assessed by MPO activity and H&E histology, and the expression of proinflammatory cytokines peak at day 2 post-LPS, and remain markedly elevated at day 7. Even at day 17 post-LPS, the inflammatory response is not completely resolved. Corn oil administration without LPS did not induce inflammatory responses as both MPO activity and expression of proinflammatory genes were unaltered compared with levels observed in lungs of corn oil-free mice at baseline. Consistently, lung injury including increases in pulmonary vascular permeability, lung edematogenesis, and lung cell death did not peak until 72hours post-challenge and remains markedly increased even at day 15 compared with oil-free mice. However, in oil-free mice, lung injury peaked as quickly as 24 hours post-LPS and fully recovered within 3 days post-LPS challenge. Cell proliferation responsible for repair also peaked at 72 hours post-LPS challenge and returned to baseline by day 5 in oil-free mice, whereas equivalent levels did not peak until day 7 in oil/LPS-challenged mice. A previous study has shown that orotracheal instillation of hydrochloric acid along with hyperoxia induces lung injury that peaks between days 1–3 post-challenge, while recovery from injury occurs over days 5–10 [25]. The prolonged time course of recovery from lung injury that is exhibited in our oil/LPS model of ARDS could facilitate studies of the mechanisms that regulate the repair processes that are crucial for re-establishment of pulmonary vascular integrity and function following sepsis challenge.

Pulmonary fibrosis is a frequent complication of ARDS and the mortality rate is much greater (approximately 60%) in ARDS patients who also develop pulmonary fibrosis [26, 27]. In human ARDS, pulmonary fibrosis appears in the interstitial lung parenchyma, e.g. in thickened alveolar septa and basement membrane [9, 28, 29]. There is little information about lung fibrosis in various experimental models of ARDS. Some evidence of pulmonary fibrosis was previously demonstrated in a mouse model of hydrochloric acid/hyperoxia-induced lung injury, but this was limited to the bronchiolar walls [25], which is likely ascribed to the damage of the bronchiolar epithelial cells in the model. Authors of this methodological study noted that the trachea must be carefully manipulated during instillation of hydrochloric acid, and that surrounding temperature and humidity must be controlled in order to prevent animal mortality. In a model of chronic inflammatory lung injury, administration of bleomycin results in early inflammation followed by fibrosis [30], but the parallels between this model and the pathogenesis/repair of lung injury in ARDS patients have been questioned [11]. Other chronic models of ARDS include i.t. administration of LPS, in which collagen deposition persists for 8 weeks [31], or i.p. administration of zymosan with or without paraffin oil [32]. In the technically simple and acute model we have characterized in the current study, prominent pulmonary fibrosis is exhibited in perivascular and peribronchial regions as well as in the interstitial lung parenchyma near medium/large-sized vessels. Intriguingly, fibrosis is initially limited to the adventitia of pulmonary vessels at day 7 post-LPS challenge, then develops in the lung parenchyma during the late recovery phase, e.g., 17 and 20 days post-LPS. Less intensive peribronchial fibrosis is also seen during the late recovery phase. But, corn oil-administration *per se* didn’t induce pulmonary fibrosis. These data suggest that slow recovery (or impaired repair) leads to development of fibrosis. Consistently, fibrosis is seen in only 3% of ARDS patients of less than one week evolution whereas 65% beyond the 3^rd^ week [28]. Severe fibrosis often occurs in ARDS patients subjected to mechanical ventilation [29, 33]. It follows that the level and distribution of fibrosis observed in our corn oil/LPS model could be exacerbated by combining the corn oil/LPS treatment regimen with mechanical ventilation.

Human ARDS is likely a result of multiple interactions between host-inherent factors (e.g. genetic determinants), primary risk factors (e.g. sepsis), and co-existing diseases (e.g. diabetes). Although any single mouse model of ARDS is unlikely to fully reproduce the pathobiology of ARDS found in man, the addition of corn oil to LPS challenge appears to move the well-used LPS-induced animal model of ARDS closer to the pathological scenario found in ARDS patients. At the very least, our model provides an improved means of investigating temporal mechanisms that regulate the initial inflammatory and injurious response, the subsequent restoration of endothelial barrier function and vascular repair, and the ultimate resolution of inflammatory injury following sepsis, as well as potential mechanisms of lung fibrosis associated with ARDS. In addition, our novel model could be developed furthermore by combining corn oil/LPS treatment with mechanical ventilation, i.e. in a so-called “2-hit” scenario that may be more clinically relevant than currently available models [34].

In conclusion, the corn oil/LPS mouse model we present here reproduces multiple pathological features of human ARDS, some of which have not been extensively shown in other animal models of ARDS (i.e. prolonged time course of injury and recovery, and widespread fibrosis). This novel model of sepsis-induced lung injury could ultimately facilitate the development of novel treatments for patients with ARDS.

## Supporting information

S1 File ARRIVE Guideline Checklist. (PDF)
